# Differences in Swallowing between High and Low Concentration Taste Stimuli

**DOI:** 10.1155/2014/813084

**Published:** 2014-04-30

**Authors:** Ahmed Nagy, Catriona M. Steele, Cathy A. Pelletier

**Affiliations:** ^1^Toronto Rehabilitation Institute, University Health Network, 550 University Avenue, No. 12-101, Toronto, ON, Canada M5G 2A2; ^2^University of Fayoum, Fayoum 63514, Egypt; ^3^University of Toronto, Toronto, ON, Canada M5V 1G7; ^4^Bloorview Research Institute, Toronto, ON, Canada M4G 1R8; ^5^University of Arkansas for Medical Sciences, Little Rock, AR 72205, USA; ^6^Charlestown Retirement Community, Catonsville, MD 21228, USA

## Abstract

Taste is a property that is thought to potentially modulate swallowing behavior. Whether such effects depend on taste, intensity remains unclear. This study explored differences in the amplitudes of tongue-palate pressures in swallowing as a function of taste stimulus concentration. Tongue-palate pressures were collected in 80 healthy women, in two age groups (under 40, over 60), stratified by genetic taste status (nontasters, supertasters). Liquids with different taste qualities (sweet, sour, salty, and bitter) were presented in high and low concentrations. General labeled magnitude scale ratings captured perceived taste intensity and liking/disliking of the test liquids. Path analysis explored whether factors of taste, concentration, age group, and/or genetic taste status impacted: (1) perceived intensity; (2) palatability; and (3) swallowing pressures. Higher ratings of perceived intensity were found in supertasters and with higher concentrations, which were more liked/disliked than lower concentrations. Sweet stimuli were more palatable than sour, salty, or bitter stimuli. Higher concentrations elicited stronger tongue-palate pressures independently and in association with intensity ratings. The perceived intensity of a taste stimulus varies as a function of stimulus concentration, taste quality, participant age, and genetic taste status and influences swallowing pressure amplitudes. High-concentration salty and sour stimuli elicit the greatest tongue-palate pressures.

## 1. Introduction

The influence of taste on swallowing behavior is not well understood, although several studies in the literature point to the possibility that particular taste stimuli may have the potential to improve swallowing function in individuals with dysphagia. To date, sour tasting stimuli such as lemon juice or citric acid solutions have been most frequently studied [1–7]. A critical question arising from these studies is whether the influence of taste on swallowing is concentration dependent [2]. A study by Pelletier and Lawless [2] found that a high-concentration (2.7% w/v) sour citric acid solution reduced penetration aspiration in older adults undergoing endoscopic examination of swallowing, but lower-concentration sour and sweet-sour stimuli did not have this effect. The authors interpreted these results to suggest that high-concentration sour stimuli might activate trigeminal nerve afferent receptors and influence swallowing via a phenomenon called chemesthesis [2].

Very few studies have investigated the impact of varying stimulus concentration on swallowing physiology. In a study on rats by Kajii and colleagues [8], the efficiency of reflex swallow initiation after applying sour and other taste solutions to the pharyngolaryngeal mucosa was investigated. Acetic and citric acids, both of which are sour, showed the highest efficiency in eliciting the reflex swallow, and this efficiency increased at higher stimulus concentrations. These data have been interpreted to suggest that higher-concentration sour stimuli may alter response thresholds at the oropharyngeal receptor level, yielding heightened stimulation of swallowing trigger neurons in the nucleus tractus solitarius, which in turn facilitate heightened activation of swallowing motor neurons via the nucleus ambiguus, ultimately resulting in more efficient swallowing [2]. In an electromyography study by Palmer and colleagues [5], sour lemon juice stimuli were shown to elicit stronger contraction of the suprahyoid musculature, as well as a muscle contraction pattern that was more tightly concatenated across the onsets of the mylohyoid, geniohyoid, and anterior belly digastric muscles compared to water. Thus, taste appears to possibly modulate both the timing and strength of swallowing.

High-concentration stimuli are usually perceived to be of greater intensity than lower concentrations of the same stimuli [9]. However, the perceived intensity for a given stimulus may differ across individuals, based on factors including prior experience, age, gender, disease, and (in the case of taste perception) genetic taste status [10]. Taste sensitivity is known to vary across the population based on genetic histoanatomical differences on chromosome 7: individuals with two recessive alleles of the taste gene are nontasters, those who have a dominant and recessive combination are medium tasters, and those with two dominant alleles are genetic* supertasters*. Supertasters are known to report heightened perception of bitterness when tasting 6n-propylthiouracil (PROP), and the degree of bitterness perceived is reported to be proportional to the density of fungiform papillae on the tongue [11].

The perceived intensity of a stimulus differs from its palatability, which reflects personal preference on the part of the person exposed to the stimulus. Only one study has explored the relationship between taste stimulus palatability and swallowing behaviors, measured in the form of tongue-palate pressures [3] and found no effect. More recently, Dietsch and colleagues [12] have shown that the addition of barium to taste stimuli lowers both their perceived intensity and palatability, with these effects being seen more strongly in older participants and in supertasters. Thus, the incorporation of both palatability and perceived intensity measures when studying the influence of taste and taste concentration on swallowing is warranted. This was the goal of the current study, which forms part of a larger project known as the Arkansas Taste and Swallowing Study (ARTSS). Specifically, the analysis described in this paper sought to answer the following questions using a sample of healthy adult women with divergent age profiles and genetic taste status and taste stimuli with four perceptual taste qualities (sweet, sour, bitter, and salty), each serving in two concentrations (high, low).

(1a) Does perceived taste intensity vary as a function of taste quality, stimulus concentration, age group, and/or genetic tasting status?

For this question, we hypothesized that greater perceived taste intensity would be reported by all participants for higher stimulus concentrations and that supertasters would report higher perceived taste intensity than nontasters.

(1b) Do palatability ratings vary as a function of taste quality, stimulus concentration, age group, and/or genetic tasting status?

We expected that the low-concentration stimuli would be rated as more palatable than high-concentration stimuli by all participants and that the sweet stimuli would be preferred (i.e., rated as more palatable) than the sour, salty, and bitter stimuli. We also expected to see an effect of genetic taste status, such that palatability ratings would be more extreme (i.e., greater like/dislike) in supertasters.

(2) Do strength-normalized measures of anterior tongue-palate swallowing pressure vary as a function of taste quality, stimulus concentration, age group, and/or genetic taste status?

Our hypotheses were that stronger amplitudes of tongue-palate pressure would be seen for the sour stimuli compared to the other taste qualities and also for the high-concentration stimuli in comparison to the low concentration stimuli.

(3a) Does perceived taste intensity modulate the effects seen in question 3?
(3b) Does palatability modulate the effects seen in question 3?

With respect to the predicted modulatory effects of palatability and perceived intensity on tongue-palate pressure variation, we adopted the null hypothesis, expecting no modulatory effects of palatability or perceived intensity on tongue-pressure amplitudes. However, should modulatory effects be identified, we would expect to see greater modulation in supertasters given the predictions of higher taste intensity ratings and more extreme palatability ratings in supertasters.

## 2. Methods

The methods of the ARTSS study have been reported elsewhere [13] but will be briefly summarized below.

### 2.1. Participants

A sample of 80 healthy community dwelling women was recruited, stratified by genetic taste status, based on their bitterness ratings (≤20 versus ≥50) of a PROP filter paper using the general labeled magnitude scale (gLMS) [14]. The study sample was limited to women given that females are known to be more likely to classify at the extremes of the genetic taste distribution than males [15]. Genetic taste status was equally distributed (20 supertasters, 20 nontasters) in each of two age cohorts (young: <40; mature: ≥60 years of age). Participant demographics are summarized in Table 1. All participants were deemed to have adequate cognitive ability to participate based on a score of at least 25 on the minimental state examination [16]. A total of 222 women were screened for inclusion in order to accrue the desired sample size with the planned stratification of genetic taste status within age group.

### 2.2. Stimuli

Pure taste stimuli (i.e., without aroma) were prepared in deionized water (Millipore 60 LiterProgrardTM Tank), with paired concentrations (high and low) for each of four taste qualities (sweet, sour, bitter, and salty).  Table 2 lists the chemicals used and their molar concentrations for each stimulus. Additional water, barium, ethanol, and carbonated stimuli were also part of the protocol [12, 17] but were only presented at single concentrations and are therefore not included in the analysis discussed in this paper. Given that the focus of this paper is on the influence of perceptual characteristics of the taste stimuli (intensity and palatability), we adopt the convention of referring to the stimuli by their taste qualities rather than their chemical compositions.

### 2.3. Protocol

Stimulus sets of chilled 5 mL samples of each test liquid were prepared in clear cups and arranged in a random order. All samples were kept in a refrigerator until presentation. Tongue-pressure sensors were applied (described below), together with a nasal cannula (to record airflow direction) and submental surface electromyography sensors. When the participants had acclimatized to the sensors (approximately 5 minutes), each randomized sample was swallowed and rated for taste intensity using the gLMS [14]. Participants were blinded to the content of each stimulus prior to sampling, and 3 or more oral rinses with room-temperature tap water were used after each sample until the participant confirmed that they no longer had any residual perception of taste or mouthfeel. After completion of the taste intensity ratings for the entire stimulus set, the sensors were removed and a 15–30 minute break was taken before proceeding with the second part of the experiment. Participants remained in the data collection room throughout the break and were not allowed to eat or drink during that time. For the second part of the experiment, participants swallowed each randomized sample again, with postsample rinses employed as previously described to remove residual taste or mouthfeel. Palatability ratings (i.e., degree of like/dislike) were captured using a hedonic version of the gLMS (H-gLMS) [18]. The H-gLMS resembles two mirrored and stacked gLMS scales such that the range is −100 to +100, reflecting a range from intense dislike to intense like.

### 2.4. Tongue-Palate Pressure Measurement

In order to monitor swallowing function, three types of data (respiratory, tongue-palate pressure, and submental electromyography) were collected using the KayPENTAX Digital Swallow Workstation (Montvale, NJ). Only the tongue-palate pressure will be discussed here. The three-bulb lingua-palatal sensor array provided with the KayPENTAX Swallowing Signals Lab was adapted for the study by removing one bulb from the pressure sensor strip; this was done to avoid a biased sample population given experience that female participants with smaller mouths frequently gag with the full-length strip [3]. The shorter two-bulb sensor strip was secured with a medical adhesive (Stomahesive, Convatec, St. Laurent, Quebec) to the roof of the mouth in midline, with the anterior bulb positioned immediately behind the upper teeth. Given previous evidence that data registered at the mid palate are highly volatile [19], perhaps due to variations in palatal vault height across participants, the current analysis is restricted to data collected at the anterior palate. Pressures were measured continuously within a range of 0–500 mmHg at 250 Hz. Time was allowed for acclimatization to the sensors prior to beginning swallowing tasks.

### 2.5. Data Processing

**Figure 1:**
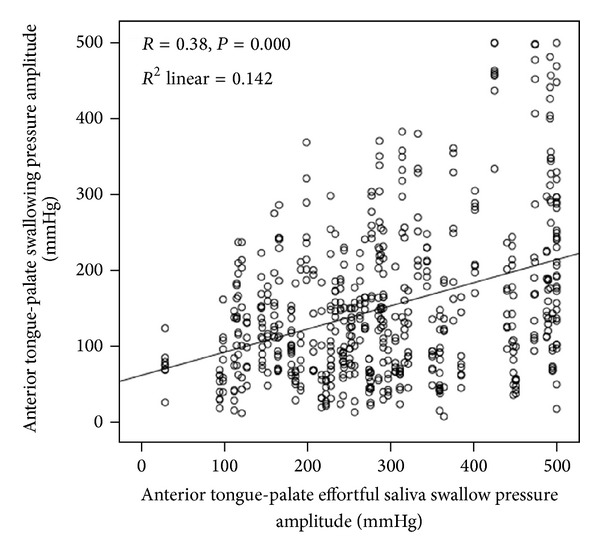
Relationship between tongue-palate pressures observed during liquid swallowing tasks and participant strength, measured as peak tongue-palate pressure during an effortful saliva swallowing task.

The anterior palate pressure bulb signal was indexed using the KayPENTAX DSW software cursor function to find and extract amplitude measures of the onsets and peaks of pressure for each swallow in the protocol. Before proceeding further, we explored the possibility that measures of tongue pressure amplitude in this data set might vary according to differences tongue strength across participants. Pearson's correlations between the swallowing pressure data and a reference measure of each participant's peak amplitude collected during an effortful saliva swallow task showed a relationship of* r* = 0.38,* P* = 0.000. Linear regression showed that 14.2% of the variability in swallowing pressure amplitudes was explained by variations in amplitude on the effortful saliva swallow task (Figure 1). Therefore, we decided to transform all swallowing pressure amplitude measures to strength-normalized values, expressing them as a percent of the effortful saliva swallow strength reference [20].

## 3. Analysis

### 3.1. Statistics

**Figure 2:**
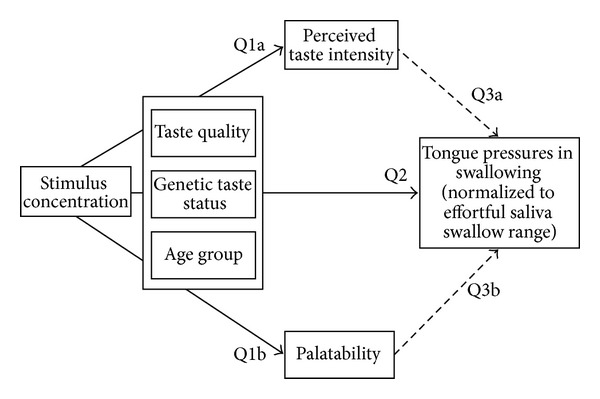
Diagram illustrating the path analysis used in this study.

Path analysis (see Figure 2) was used [21] to address the questions in this study. Path analysis is a well-established statistical approach in which a stepwise series of ANOVAs are performed to test components before building a final AN(C)OVA model. Path analysis begins with the initial assumption that there may be modulatory effects of covariate factors on the patterns of variation seen in a dependent variable. The approach first tests whether these potential covariates vary as a function of the main factors in the model (in this case, questions (1a) and (1b), exploring whether taste intensity or palatability varies as a function of taste quality, stimulus concentration, genetic taste status, and age group). Based on the results of these preliminary explorations, the argument for exploring the influence of these potential covariates on the main dependent variable at a later stage in the analysis (questions (3a) and (3b)) is either supported or refuted. Question (2) in our paper describes the primary study question (differences in tongue-pressure amplitudes as a function of taste quality, stimulus concentration, genetic taste status, and age group) without consideration of the covariates. The final step in the model refines question (2) model by integrating significant factors from questions (1a) and (1b) as covariates (in this case, questions (3a) and (3b)). Each step in this process involved fully factorial mixed-model analyses of variance with a compound symmetry structure, which was determined to have the best fit with the data based on restricted log likelihood estimation. For questions (1a), (1b), and (2) these were 4-way ANOVAs with within-participant factors of taste quality and stimulus concentration and between-participant factors of genetic taste status and age group. For questions (3a) and (3b), the modulatory effects of taste intensity and palatability were explored separately by adding these factors as covariates into the statistical model (ANCOVA). For all analyses, an a priori alpha criterion for statistical significance was established at *P* < 0.05. Significant effects and interactions were further explored using Sidak tests for pairwise comparisons, with the strength of these pairwise significant differences being further characterized using Cohen's* d* measures of effect size (0.2–0.5 = small; 0.5–0.8 = medium; >0.8 = strong) [22].

## 4. Results

### 4.1. Question (1a) (Taste Intensity)

**Figure 3:**
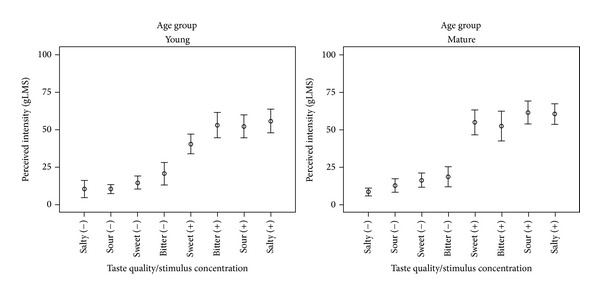
Three-way interaction observed for ratings of perceived intensity between stimuli with four taste qualities as a function of stimulus concentration and participant age group.

Table 3 provides descriptive statistics for the perceptual ratings of taste intensity by taste quality, stimulus concentration, age group, and genetic taste status. Several 2-way interactions were found to be significant: age group X stimulus concentration (*F*(1, 454.15) = 4.215,* P* = 0.041), genetic taste status X stimulus concentration (*F*(1, 454.15) = 17.639,* P* = 0.000), and taste quality X stimulus concentration (*F*(3, 451.18) = 9.096,* P* = 0.000). Additionally, statistically significant main effects were found for stimulus concentration (*F*(1, 454.15) = 918.117,* P* = 0.000, Cohen's* d* = 1.38, i.e., large), and genetic taste status (*F*(1, 74.75) =13.278,* P* = 0.000, Cohen's* d* = 0.41, i.e., small). These results can be summarized as showing that ratings of greater perceived intensity were triggered by higher stimulus concentrations and that supertasters reported greater perceived intensity than nontasters. These findings provide justification for exploring the modulatory effects of taste intensity at a later stage in the analysis process (question (3a)). The results for question (1a) are illustrated in Figure 3 with the low and high concentrations of each taste quality marked by (−) and (+) diacritics, respectively. As shown in the figure, the interactions of stimulus concentration, age group, and genetic taste status reveal a heightened degree of increase in perceived intensity for the high concentration stimuli in the older participants (Cohen's* d* = 0.36, i.e., small) and in supertasters (Cohen's* d* = 0.61, i.e., medium). Effect size measures for the concentration comparison were large for all four taste qualities with weaker contrasts seen for the sweet (*d* = 1.13) and bitter stimuli (*d* = 1.16) than for the sour (*d* = 1.57) and salty (*d* = 1.66) stimuli. Interestingly, the low- and high-concentration salty stimuli accounted for both the lowest and highest reported intensity ratings and thus formed the boundary conditions across all stimuli tested.

### 4.2. Question (1b) (Palatability)

Table 4 shows descriptive statistics for ratings of palatability by taste quality, stimulus concentration, age group, and genetic taste status. In general, it can be noted that each taste quality was either liked (sweet) or disliked (sour, salty, or bitter), and the degree of liking or disliking was dependent on perceived intensity. Explorations of this relationship using Pearson's correlations and absolute values of the palatability ratings (i.e., removing the polarity) showed a correlation of* r* = 0.63 (*P* = 0.000) with perceived intensity. Several significant results support the plan to explore the modulatory effects of palatability in question (3b) of the analysis.

Two different 3-way interactions proved significant for the palatability parameter. First, stimulus concentration showed a significant 3-way interaction with age group and taste quality (*F*(3, 467.60) = 3.122,* P* = 0.026), building on significant 2-way interactions between stimulus concentration X age group (*F*(1, 468.4) = 10.104,* P* = 0.002), stimulus concentration X taste quality (*F*(3, 467.60) = 30,* P* = 0.000), and age group X taste quality (*F*(3, 468.15) = 3.27,* P* = 0.021). Second, a significant taste quality X age group X genetic taste status interaction was seen (*F*(3, 468.145) = 4.726,* P* = 0.003), building on the previously reported significant 2-way taste quality X age-group interaction. To understand these interactions, it is important first to dissect their components in terms of significant main effects. Statistically significant differences in palatability were found between the different taste qualities (*F*(3, 468) = 146.43,* P* = 0.000), with the bitter stimuli being significantly more disliked than the sweet stimuli (Cohen's* d* = 1.55, i.e., large) and also more disliked than the salty and sour stimuli (*d* = 0.33 to 0.34, i.e., small). In addition to being markedly preferred over the bitter stimuli, the sweet stimuli were also significantly more liked than the salty and sour stimuli (Cohen's* d* = 1.2, i.e., large). Significant differences in palatability were also found as a factor of stimulus concentration: high-concentration stimuli were liked/disliked significantly more than the corresponding low-concentration stimuli ((*F*(1, 468) = 88.17,* P* = 0.000, Cohen's* d* = 0.53, i.e., medium). Furthermore, supertasters reported more extreme like/dislike than nontasters (*F*(1, 68.13) = 6.22,* P* = 0.015, Cohen's* d* = 0.19, i.e., small).

**Figure 4:**
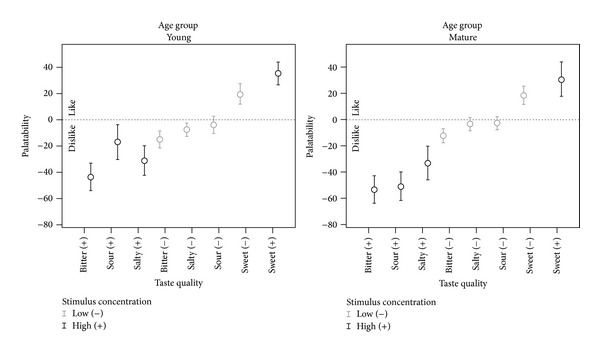
Three-way interaction observed for palatability ratings of liquid stimuli as a function of taste quality, stimulus concentration, and participant age group.

**Figure 5:**
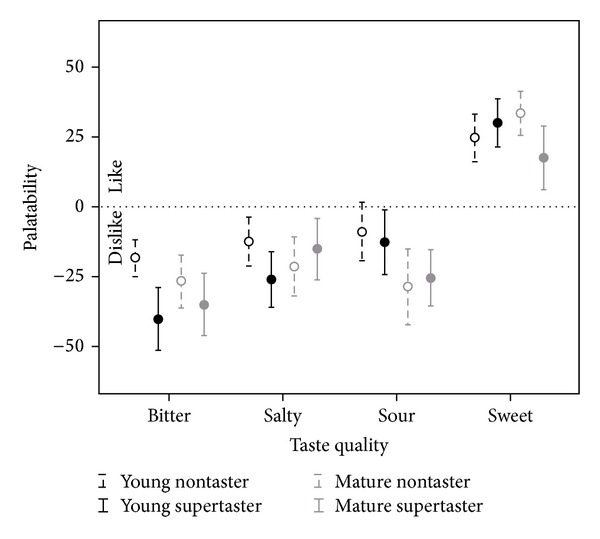
Three-way interaction observed for ratings of palatability for liquid stimuli as a function of taste quality, participant age group, and genetic taste status.

The combined results arising from the interactions of stimulus concentration, age group, and taste quality are illustrated in Figure 4 and can be summarized as showing the strongest liking for the high-concentration sweet stimulus, the greatest dislike for the high concentration bitter stimulus, and a difference between younger and older participants with respect to whether the high-concentration sour was more disliked than the high-concentration salty stimulus (or vice versa). The low concentration sweet stimulus was more liked than the other three low-concentration stimuli, for which palatability ratings were in the mild dislike range, close to the neutral zero value. Similarly, the combined effect arising from the interaction of taste quality X age group X genetic taste status interaction is illustrated in Figure 5, which shows overall liking for the sweet taste quality and disliking for the other three tastes (of which the bitter was most disliked). Among the supertasters, the mature participants showed less dislike for the bitter and sour stimuli and less liking of the sweet stimuli than the younger participants but greater disliking of the salty stimulus. Among the nontasters, the mature participants showed more extreme liking or disliking for all four tastes than the younger participants.

### 4.3. Question (2) (Tongue Pressure Amplitudes)

**Figure 6:**
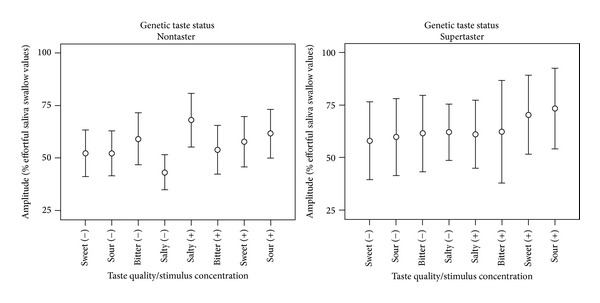
Differences in strength-normalized measures of tongue-palate pressure amplitudes as a function of stimulus taste-quality and concentration in genetic nontasters and supertasters.

Table 5 provides descriptive statistics for the strength-normalized values of anterior tongue-palate swallow pressure amplitude. A significant 3-way taste quality X stimulus concentration X genetic taste status interaction was found (*F*(3, 476) = 3.77,* P* = 0.011) together with a significant 2-way taste quality X stimulus concentration interaction (*F*(3, 476) = 2.76,* P* = 0.04). Additionally, a significant main effect of stimulus concentration was found (*F*(1, 476) = 13.48,* P* = 0.000) but with only a negligible effect size (Cohen's* d* = 0.17), showing larger amplitudes of tongue pressure for higher-concentration stimuli. The 3-way interaction is illustrated in Figure 6, showing a general pattern of stronger tongue-palate pressures for higher-concentration stimuli in the supertaster group. A marked increase in pressure amplitude is also seen for the high-concentration salty stimulus compared to its low concentration comparator amongst nontasters.

### 4.4. Question (3) (Modulation of Tongue Pressure Variation related to Taste Intensity and Palatability)

Given the results of questions (1a), (1b), and (2), continued explorations of the statistical model incorporating covariate effects of perceived intensity and palatability were warranted. The model was simplified to remove age group as a factor given the absence of age-group differences (or interactions involving age-group contrasts) in strength-normalized measures of anterior tongue-palate pressure amplitudes in question (2).

The incorporation of perceived intensity into the model as a covariate (i.e., question (3a)) yielded a single significant finding in the form of a significant 3-way interaction between taste quality, stimulus concentration, and genetic taste status (*F*(3, 403.91) = 3.44,* P* = 0.017). It is noted that this finding matches the 3-way interaction pattern observed previously in question (2). Intensity by itself did not show significance as a factor.

The incorporation of palatability ratings into the model as a covariate (i.e., question (3b)) yielded a single significant main effect of stimulus concentration on strength-normalized measures of tongue-palate pressure amplitude (*F*(1, 413) = 12.271,* P* = 0.001) with stronger pressures generated for higher-concentration stimuli. This finding also matches the results seen previously in question (2). Palatability by itself did not show significant influence.

## 5. Discussion

This study explores variations in tongue pressure amplitudes during the swallowing of different tastant solutions prepared in both high and low concentrations. The results confirm that high concentrations of the four-taste qualities studied (sweet, sour, salty, and bitter) elicit stronger swallowing pressures than lower concentrations. This effect is heightened for salty stimuli and in individuals who are genetic supertasters. Additionally, by exploring both the main and modulatory effects of perceived taste intensity and palatability on swallowing pressures, this study confirms that perceived taste intensity influences the effects seen across stimulus concentration and taste quality in supertasters compared to nontasters. Supertasters report heightened taste intensity experience compared to nontasters and therefore show greater intensity-based modulation in swallowing pressures. Additionally, differences in reported palatability for these stimuli were seen in older versus younger adults as a function of taste quality and stimulus concentration, as well as genetics, and these differences were carried through to swallowing pressure amplitudes. Specifically, older adults reported greater dislike for a high intensity sour stimulus than younger participants. Neither taste intensity nor palatability had a direct influence on the amplitudes of tongue pressure used when swallowing the test stimuli.

This is not the first time that researchers have explored variations in tongue-palate pressure amplitudes during the swallowing of taste stimuli with differing concentrations. Pelletier and Dhanaraj [3] have previously used a similar methodology and reported that a 0.15 M sucrose stimulus and 1 M salt and citric acid stimuli elicited higher lingual pressure amplitudes compared to water. Their study used a 9-point hedonic rating scale and showed that while palatability did not have a direct influence on lingual pressures, incorporation of palatability as a covariate in their model did lessen the effect of taste quality.

The results of the current study add to the information previously reported by Pelletier and Dhanaraj [3] and are largely in agreement with our hypotheses. Our study looked more closely at the effects of stimulus concentration for sweet, sour, salty, and bitter stimuli without reference to water. The concentrations of our stimuli were more extreme in both the low- and high-concentration directions than those used in the Pelletier and Dhanaraj study [3], with the exception of the high-concentration sour stimulus, which was 0.128 M in both studies. We added considerations of genetic taste status and measures of perceived taste intensity and were able to confirm that intensity is perceived to be greater for higher concentrations of taste stimuli and that this difference is noticed more markedly in supertasters. Further, we confirmed that palatability ratings show stronger liking for sweet stimuli than for sour, salty, or bitter stimuli and that relative liking or disliking of a stimulus is dependent on its concentration. Our use of the hedonic gLMS scale versus the 9-point hedonic rating scale used by Pelletier and Dhanaraj [3] allowed greater discrimination of palatability given its design. With respect to tongue-palate pressure amplitudes seen in swallowing, we confirmed that higher concentrations of taste stimuli elicit stronger tongue pressures. Consistent with our predictions and expanding on the young-adult data previously reported by Pelletier and Dhanaraj [3], no age-group differences were seen in swallowing pressure amplitudes. A novel finding from this study is the fact that high-concentration salty stimuli were rated as having the strongest perceived intensity (regardless of genetic taste group) and elicited the strongest tongue-palate pressure amplitudes in genetic nontasters. Our prediction that the sour stimuli would facilitate greater tongue pressures than the other stimuli was only true for the supertaster subgroup (Figure 6). With the exception of the Pelletier and Dhanaraj study [3], we are not aware of prior studies in which both sour and salty stimuli have been studied together, and hence this study opens the door to exploration of taste stimuli beyond sour in terms of the influence of taste quality on swallowing behaviors. Because high concentrations of salt can also stimulate a chemesthetic response in trigeminal nerve receptors similar to the response seen with high concentrations of citric acid, it is not entirely surprising to see that increased tongue-palate pressure amplitudes were observed with the high-concentration salty stimuli [23, 24]. However, it was surprising to see this pattern in genetic nontasters versus supertasters. Nevertheless, it appears that tongue-palate pressure amplitudes increase in response to chemesthetic stimulation. This may have important implications for future studies and also for clinical applications. Our findings provide support for the idea that chemesthetic stimuli may elicit improvements in swallowing in some individuals with dysphagia. Future research will need to explore thresholds with respect to stimulus concentration that are adequate to elicit positive behavioral changes and to determine whether such responses can be achieved with stimuli that remain palatable.

Neither intensity nor palatability ratings were found to independently influence tongue-palate pressures. Nevertheless, both of these modulators were found to influence the degree to which stimulus concentration effects were seen in tongue-palate pressure amplitudes. Incorporation of intensity ratings into the model revealed an effect by which stimulus concentration interacted with taste quality and genetic taste status, while consideration of palatability led to a main effect of concentration. The combination of observed findings can be summarized as showing that stronger concentrations of taste stimuli are rated as more liked or disliked, depending on the taste quality, and elicit stronger tongue-palate pressures in swallowing. Further, supertasters are more sensitive to manipulations of stimulus concentration. However, it can also be concluded that the greatest facilitation of stronger tongue-palate pressure amplitudes occurred with stimuli that were perceived to have the greatest intensities and elicited a chemesthetic response. The high-concentration bitter and sweet stimuli, which represented the most extreme palatability ratings, did not facilitate increased tongue-palate pressures.

The findings of this study have important implications for clinicians who are considering the use of taste stimuli in treatment for dysphagia. This study adds to a growing body of the literature that suggests that the benefits of using taste stimuli may depend on whether or not the stimuli have chemesthetic properties. Additionally, our study suggests that it is relevant to know whether a person is a supertaster or a nontaster and that it may further be helpful to determine the threshold concentrations of stimuli that are needed to elicit chemesthetic response when choosing stimuli for use in treatment. It is also plausible that some patients with oropharyngeal dysphagia may present with altered sensory thresholds for taste or chemesthesis perception; in these cases, confirming that a stimulus is perceived to have a high taste intensity would be advised before recommending its use.

### 5.1. Limitations

This study is not without limitations. In particular, the study demonstrates that the high-concentration stimuli used in the experiment were unpalatable, with the exception of the sweet tastant. This presents obvious challenges in terms of applying the study results to clinical practice. We recommend that the current data be considered as a demonstration of effect, but further research will be needed to determine whether heightened swallowing pressures can be elicited with stimulus concentrations that are more palatable. Furthermore, although the study demonstrates heightened tongue-palate pressure amplitudes as a function of increased taste stimulus intensity, it is unknown whether these differences in swallowing pressures can be expected in individuals with swallowing impairment and whether they are of sufficient magnitude to yield clinically relevant differences in swallowing function, such as improved bolus propulsion and clearance.

## 6. Conclusions

In conclusion, the data show that the perceived intensity of a taste stimulus (which varies as a function of stimulus concentration, taste quality, participant age, and genetic taste status) influences tongue pressure amplitudes in swallowing. Palatability ratings, which vary as a function of stimulus concentration and differ between stimuli with different taste qualities, do not appear to influence tongue pressure amplitudes in swallowing. This finding is consistent with previous results in the dysphagia literature [3]. The high concentration sour and salty stimuli in this protocol were perceived to have the highest intensities and elicited the highest amplitudes of tongue-palate pressure. We attribute these findings to the chemesthetic properties of these stimuli.

## Figures and Tables

**Table 1:** Participant demographics.

Genetic taste status	Age group	*N*	Mean Age (years)	Standard deviation
Nontasters	Young (<40)	20	25.8	4.7
	Mature (>60)	20	71.5	8.7

Supertasters	Young (<40)	20	26.5	3.4
	Mature (>60)	20	72.6	7.4

**Table 2:** Liquid stimuli used in the study.

Taste quality	Chemical	Stimulus concentration	Molar concentration
Sweet	Sucrose (table sugar)	High (+)	1 M
		Low (−)	0.15 M

Sour	Citric acid USP*	High (+)	0.128 M
		Low (−)	0.002 M

Salty	Sodium chloride USP*	High (+)	1 M
		Low (−)	0.034 M

Bitter	Anhydrous caffeine USP*	High (+)	0.032 M
		Low (−)	0.003 M

*All chemicals were obtained from http://www.sciencelab.com/.

**Table 3:** Descriptive statistics for gLMS ratings of perceived intensity, shown by taste quality, stimulus concentration, participant age group, and genetic taste status.

Taste quality/stimulus concentration	Age group/genetic taste status	Mean	95% confidence interval		Standard deviation
			Lower boundary	Upper boundary	
Salty (−)	Young nontaster	6.44	3.18	9.71	6.57
	Young supertaster	14.06	3.17	24.94	21.89
	Mature nontaster	7.44	4.32	10.57	4.07
	Mature supertaster	9.30	5.24	13.36	5.68

Sour (−)	Young nontaster	10.35	5.56	15.14	9.31
	Young supertaster	10.28	6.66	13.89	7.27
	Mature nontaster	11.07	4.23	17.91	11.85
	Mature supertaster	14.58	8.17	21.00	10.09

Sweet (−)	Young nontaster	12.79	6.42	19.16	13.21
	Young supertaster	16.05	9.67	22.43	13.63
	Mature nontaster	14.32	7.56	21.07	14.01
	Mature supertaster	18.60	11.87	25.33	12.15

Bitter (−)	Young nontaster	16.86	6.29	27.42	18.30
	Young supertaster	24.07	12.33	35.81	21.20
	Mature nontaster	13.00	2.13	23.87	15.20
	Mature supertaster	22.43	13.66	31.20	15.18

Sweet (+)	Young nontaster	33.80	26.63	40.97	15.33
	Young supertaster	47.20	36.34	58.06	23.21
	Mature nontaster	46.25	33.65	58.85	26.93
	Mature supertaster	63.60	53.21	73.99	22.20

Bitter (+)	Young nontaster	41.42	30.65	52.20	22.36
	Young supertaster	64.05	52.38	75.72	24.94
	Mature nontaster	39.40	24.01	54.79	32.89
	Mature supertaster	66.83	57.41	76.26	18.96

Sour (+)	Young nontaster	42.20	33.36	51.04	18.88
	Young supertaster	62.15	50.58	73.72	24.73
	Mature nontaster	55.20	43.69	66.71	24.60
	Mature supertaster	67.75	57.98	77.52	20.87

Salty (+)	Young nontaster	50.26	38.31	62.21	24.79
	Young supertaster	61.00	50.19	71.81	23.10
	Mature nontaster	53.55	42.24	64.86	24.17
	Mature supertaster	67.35	59.77	74.93	16.19

**Table 4:** Descriptive statistics for H-gLMS ratings of palatability, shown by taste quality, stimulus concentration, participant age group, and genetic taste status.

Taste quality/stimulus concentration	Age group/genetic taste status	Mean	95% confidence interval		Standard deviation
			Lower boundary	Upper boundary	
Bitter (+)	Young nontaster	−28.89	−39.16	−18.62	20.66
	Young supertaster	−61.40	−76.86	−45.94	27.91
	Mature nontaster	−42.00	−56.03	−27.97	24.30
	Mature supertaster	−62.35	−76.73	−47.98	27.96

Sour (+)	Young nontaster	−12.70	−31.94	6.54	41.11
	Young supertaster	−22.00	−41.86	−2.14	41.20
	Mature nontaster	−53.32	−70.33	−36.30	35.30
	Mature supertaster	−48.22	−62.50	−33.94	28.71

Salty (+)	Young nontaster	−21.56	−37.51	−5.60	32.09
	Young supertaster	−41.29	−56.96	−25.63	30.46
	Mature nontaster	−37.06	−53.95	−20.18	31.68
	Mature supertaster	−29.74	−49.91	−9.57	41.85

Bitter (−)	Young nontaster	−7.72	−13.12	−2.32	10.86
	Young supertaster	−22.44	−33.70	−11.19	22.64
	Mature nontaster	−12.60	−21.39	−3.81	15.86
	Mature supertaster	−11.90	−18.56	−5.24	14.24

Salty (−)	Young nontaster	−3.44	−9.04	2.15	11.25
	Young supertaster	−11.67	−19.93	−3.40	16.62
	Mature nontaster	−5.88	−13.69	1.94	14.67
	Mature supertaster	−1.45	−8.41	5.51	14.87

Sour (−)	Young nontaster	−5.11	−11.47	1.25	12.78
	Young supertaster	−2.94	−14.59	8.70	23.42
	Mature nontaster	0.50	−8.94	9.94	17.72
	Mature supertaster	−5.15	−10.45	0.15	11.32

Sweet (−)	Young nontaster	17.39	6.27	28.51	22.37
	Young supertaster	21.61	10.31	32.91	22.72
	Mature nontaster	24.06	17.02	31.10	13.21
	Mature supertaster	14.00	2.72	25.28	24.09

Sweet (+)	Young nontaster	32.00	19.62	44.38	24.89
	Young supertaster	38.28	25.83	50.72	25.03
	Mature nontaster	42.88	29.55	56.20	25.00
	Mature supertaster	21.10	0.14	42.06	44.79

**Table 5:** Descriptive statistics for strength-normalized measures of anterior tongue-palate swallowing pressures (in % of effortful saliva swallow amplitudes), shown by taste quality, stimulus concentration, participant age group, and genetic taste status.

Taste quality/stimulus concentration	Age group/genetic taste status	Mean	95% Confidence interval		Standard deviation
			Lower boundary	Upper boundary	
Sweet (−)	Young nontaster	49.81	34.53	65.09	30.73
	Young supertaster	60.46	38.82	82.10	40.60
	Mature nontaster	54.70	37.13	72.28	36.46
	Mature supertaster	56.27	25.53	87.01	63.77

Sour (−)	Young nontaster	44.56	31.10	58.03	27.07
	Young supertaster	60.40	38.05	82.76	41.95
	Mature nontaster	59.81	43.20	76.42	34.47
	Mature supertaster	59.73	29.64	89.82	62.43

Bitter (−)	Young nontaster	52.26	37.98	66.54	28.71
	Young supertaster	69.84	45.06	94.62	46.50
	Mature nontaster	65.63	44.49	86.77	43.86
	Mature supertaster	54.85	27.18	82.52	57.41

Salty (−)	Young nontaster	43.78	32.42	55.13	22.83
	Young supertaster	69.60	49.44	89.77	37.84
	Mature nontaster	42.74	29.83	55.65	26.79
	Mature supertaster	56.23	37.34	75.12	39.19

Salty (+)	Young nontaster	68.57	50.82	86.32	35.69
	Young supertaster	65.52	44.60	86.43	39.25
	Mature nontaster	67.48	47.54	87.42	41.37
	Mature supertaster	57.88	32.34	83.42	52.99

Bitter (+)	Young nontaster	49.85	33.40	66.30	33.08
	Young supertaster	61.01	44.49	77.53	31.01
	Mature nontaster	57.87	40.61	75.13	35.81
	Mature supertaster	63.72	18.79	108.65	93.22

Sweet (+)	Young nontaster	55.28	37.13	73.44	36.51
	Young supertaster	80.46	54.56	106.37	48.61
	Mature nontaster	60.10	42.49	77.70	36.53
	Mature supertaster	62.21	33.72	90.70	59.10

Sour (+)	Young nontaster	63.29	48.54	78.04	0.00
	Young supertaster	74.08	51.85	96.31	41.72
	Mature nontaster	60.02	41.04	78.99	39.37
	Mature supertaster	73.26	41.60	104.92	65.68
